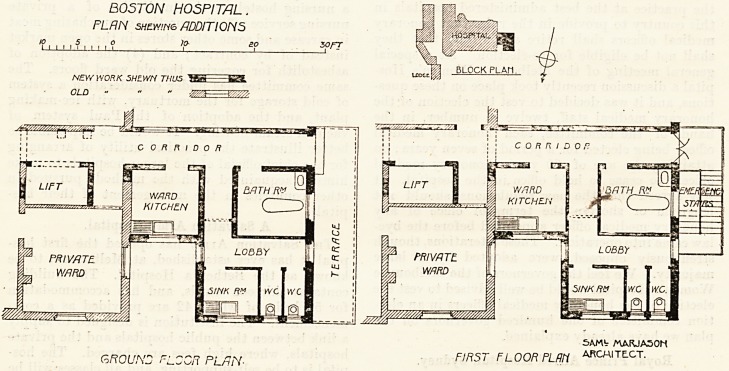# New Sanitary Wing at the Boston Hospital

**Published:** 1906-07-28

**Authors:** 


### NEW SANITARY WING AT THE BOSTON HOSPITAL.

This addition to the hospital at Boston was opened in December 1905 by the Mayor, Mr. Barron Clark. The wing is attached to the north-west corner of the old building, and, although supplying a present want, has also been designed with a view to future extension of the hospital. It is a two-story annexe; and, on entering the new corridor from the old building, a lift is found on the right-hand side, then a good ward kitchen, and further on is a bath-room. Between the kitchen and the bath-room are the sink and the closets, which are carefully separated from the other parts, and have a wide cross ventilated lobby. The first floor is exactly similar. In addition to the above-named rooms there is an emergency staircase placed at the western extremity of the block.

The annexe is small, but it is well arranged, and it evidently possesses plenty of light and ample means for the circulation of fresh air.

The exterior of the new building harmonises with that of the old one. The inside walls are lined with glazed tiles and brick dadoes; and at the junction of the wall and floor the tiles are concave so as to prevent accumulation of dust, and to render cleaning more easy to ensure. The floors of the rooms are of encaustic tile paving, and those of the corridors are laid down with terrazzo. The fittings of the baths, sinks, and closets are of the most approved kind. Fresh air inlets are provided, and provision is also made for the extraction of vitiated air. The annexe is warmed by hot-water radiators.

The architect was Mr. S. Marjason, the contractors were Messrs. Parker and Son, and the lift was supplied by Messrs. Waygood. The cost of the whole work was about £1,000.

## Figures and Tables

**Figure f1:**